# Semi-constrained posterior stabilized total knee arthroplasty reproduces natural deep knee bending kinematics

**DOI:** 10.1186/s12891-020-3059-1

**Published:** 2020-02-17

**Authors:** Takanobu Sumino, Tetsuya Tomita, Kazuomi Sugamoto, Takaharu Yamazaki, Ken Okazaki

**Affiliations:** 1 Department of Orthopaedic Surgery, Nihon University School of Medicine, 30-1 Ohyaguchikamichou, Itabashi, Tokyo, 173-8610 Japan; 2 Department of Orthopedic Biomaterial Science, Osaka University Graduate School of Medicine, 2-2 Yamadaoka, Suita, Osaka, 565-0871 Japan; 3 Department of Information Systems, Saitama Institute of Technology, 1690 Fusaiji, Fukaya, Saitama, 369-0293 Japan; 4 Department of Orthopaedic Surgery, Tokyo Women’s Medical University, Shinjuku City, Japan

**Keywords:** Total knee arthroplasty, Semi-constrained posterior stabilized system, Kinematics, Post-cam engagement, Deep knee bending

## Abstract

**Background:**

The Flexible Nichidai Knee Posterior Stabilized (FNK-PS) system was designed to provide relatively high varus-valgus stabilities without the stem extensions to patients with severe knee joint disorders. This is a combination of a large tibial post and high femoral cam adapted to a PS system. The aim of our study was to analyze the in vivo two-dimensional/three-dimensional registration kinematics of the FNK PS-total knee arthroplasty (TKA) system during deep knee bending.

**Methods:**

Nineteen knees from 15 total knee arthroplasty (TKA) patients who were able to squat with enough knee flexion were selected. During deep knee bending under weight bearing (WB) and non-weight bearing (NWB) conditions, we quantified range of motion, axial rotation, femoral anteroposterior translation, and post-cam engagement angle.

**Results:**

The maximum-flexion was significantly different between the two conditions. The mean axial femoral external rotation was 4.8° and 6.2° under WB and NWB conditions, respectively, at 120° flexion. Anteroposterior translation based on bicondylar posterior roll-back patterns was noted with increasing knee flexion. Both the medial and lateral femoral aspects were significantly more posterior during early to mid-flexion. Initial post-cam engagement occurred significantly earlier during flexion under NWB than under WB conditions. Under WB, the timing of the post-cam engagement correlated with the maximum flexion .

**Conclusions:**

The kinematics of the semi-constrained PS system reproducibly exhibited a mild external rotation with smooth posterior roll-back. This was assisted by the engagement of the large tibial post and high femoral cam during the early phase of flexion.

## Background

Constrained condylar TKA (CCK) system is recommended for patients having an unstable knee with severe deformity, medial collateral ligament (MCL) deficiency, and inadequate soft tissue balance during flexion and extension. Although constrained condylar implants provide reliable clinical outcomes [1, 2], these implants use modular stem extensions for both tibial and femoral components, which sometimes cause technical difficulties, increase the risk of large bone stock deficiencies, and affect the implant longevity [3, 4].

As an alternative to the CCK system, the Flexible Nichidai Knee (FNK) system (Nakashima Medical, Japan) was designed to give a relatively high varus-valgus stability without the stem extensions. It features a combination of a large tibial post and a high femoral cam, i.e., it is a semi-constrained PS system. These features make the FNK system useful for patients with severe knee deformities and moderate MCL deficiencies. This system shows a good postoperative recovery of the quadricep and hamstring power and has good long-term clinical outcomes and survival rates [5, 6].

Deep knee bending is an important motion in daily activities and is correlated with clinical outcomes, especially in the Asian population [7]. However, a meta-analysis of the standard PS-TKA in this population revealed that significant improvement regarding deep knee bending is not always achieved [8]. Several studies using motion capture methods for the in vivo evaluation of knee kinematics in patients with PS-TKAs suggest that the external rotation of the femur relative to the tibia is important to perform deep knee bending [9–13]. However, the PS system with a large post, adopted in the CCK and semi-constrained TKA, may interfere with the axial rotation during flexion. Deshmukh et al. reported that a non-stemmed CCK for the same femoral component, Genesis II (Smith & Nephew, USA), allowed a constrained valgus-varus motion within 2° to 3° [14]. They defined this as a semi-constrained PS-TKA and reported substantial postoperative short-term results that were comparable to those of a standard PS implant. However, none of the previous studies have analyzed the effects of a large tibial post and high femoral cam engagement on the kinematics of a semi-constrained PS-TKA, which is the main feature of the FNK system. Although this system exhibited a good range of motion, the effect of this post and cam design on the axial rotation during deep knee bending needs to be elucidated.

Therefore, the aim of our study was to use in vivo fluoroscopy to quantify the relative motion between the femoral and tibial components and the angle of post-cam engagement of the semi-constrained system during deep knee bending under weight-bearing (WB) and non-weight-bearing (NWB) conditions.

It was hypothesized that the FNK-PS system reproduces the kinematic developmental concept, having a moderate internal-external rotation during deep knee bending, and demonstrates an early post-cam engagement to assist an efficient femoral roll-back.

## Methods

### Study group

Nineteen knees from 15 female Japanese patients who had undergone TKA using the FNK-PS implant and were able to squat with enough knee flexion under WB condition were included in this study. The mean ± standard deviation age of these patients was 72.3 ± 9.5 years, and the postoperative follow-up period was 23.4 ± 19.3 months. Thirteen patients had undergone TKA for the treatment of osteoarthritis and two for the treatment of rheumatoid arthritis. Four patients had undergone bilateral TKAs. All procedures performed in studies involving human participants were in accordance with the ethical standards of the institutional research committee and with the 1964 Helsinki declaration and its later amendments or comparable ethical standards. All included patients provided written consent before being admitted into the study.

Preoperatively, there were six knees with valgus alignment and 13 knees with varus alignment. Six knees had valgus alignment, with a mean femorotibial angle (FTA) of 169.5° ± 1.4° (range, 161–170°), and 13 knees had varus alignment, with a mean femorotibial angle of 190.5° ± 3.8° (range, 182–199°). The mean postoperative FTA was 172.9° ± 2.9° (range, 169–176°). In the Kellgren and Lawrence scoring system [15], all osteoarthritis cases were grade IV. In the Larsen’s scoring system [16], all the rheumatoid arthritis cases were grade IV.

The mean range of motion was 103.5 ± 20.7° (range: 60–130°), with a mean Knee Society Function Score of 44.1 ± 16.7 (range: 15–65). Postoperatively, this score improved to 90.8 ± 11.0 (range: 70–100) and the range of motion increased to 122.6 ± 9.5° (range: 105–135°). A postoperative radiographic assessment revealed that all prosthetic components were well-fixed.

### Prosthesis design

**Fig. 1:**
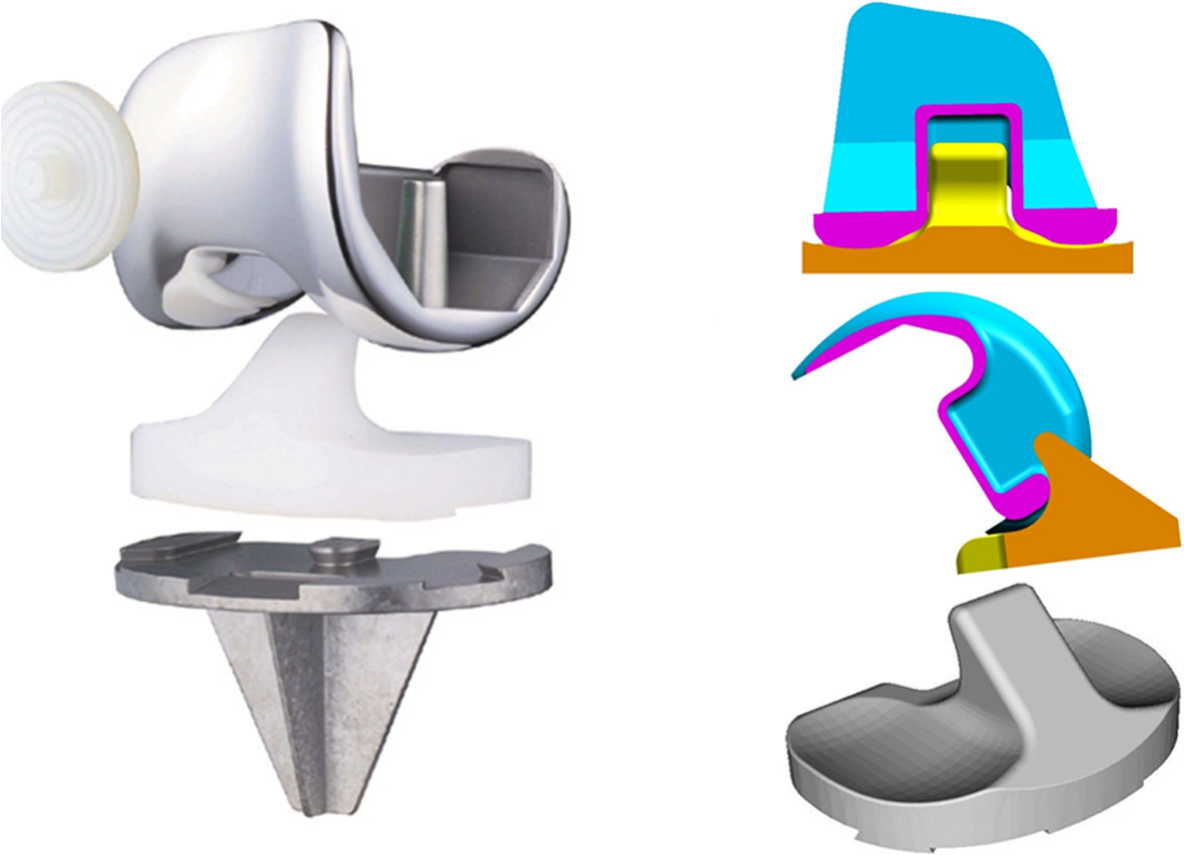
Frontal view and schemes depicting semi-constrained PS FNK with a large tibial post and high femoral cam. These images provided from Nakashima Medical, Japan

The FNK system includes a thin anterior chamber and a deep patella groove in the femoral component to reduce the pressure on the patellofemoral joint. The femoral component has a multi-radial rotation in the sagittal plane. The tibial component has a wide cross-keel to distribute the directional stress. The thinnest part of the tibial component is 3.5 mm to preserve the bone stock. The posterior constraint is provided by a “flat-on-flat” posterior cam mechanism. The anterior and posterior posts are both flat. The spine height and width were 18.8 to 23.6 mm and 11.6 to 17.8 mm, respectively, for each prosthesis size. The jumping distance ranged between 14.1 and 17.7 mm. Compared to the standard PS system, this post-cam mechanism offers a higher constraint to relative motion between the components of the TKA system (Fig. 1). It constrains the valgus-varus motion within ±2° at 0° and ± 4° at 90° of flexion, and the internal-external rotation within ±6° at 90° of knee flexion [5]. Table 1 compares the features of axial rotation and varus-valgus constraint of FNK, with other PS, CCK, and semi-constrained TKA systems. The data were obtained from a survey of four published papers [1, 5, 14, 17] and four commercial, implant websites [18–21].

**Table 1:** Degrees of Rotation and Constraint for PS TKA

	Internal-External Rotation		Varus Valgus Constraint
	Flexion 0°	Flexion 90°	
Implant Design			
Semi Constrain Implans			
FNK_PS [5]	±3°	±5°	±2° to ±4°
GenesisII constrained insert [14]	±3.5°	±3.5°	±2.5°
Vangaurd PS Plus [18]	±2°	^a^	±2°
Constrained Condylar Implants			
GenesisII revision constrained [14]	±3°	±3°	±2°
Legacy CCK [1]	±2°	^a^	±1.25°
Vangaurd 360 [19]	±0.5°	±0.5°	±1°
PFC ∑ TC3 [17]	±1.3°	±5.4°	±2.2°
Primary PS implants			
Nexgen LPS [20]	±12°	^a^	±7.5°
Vangaurd PS [18]	±15°	±15°	–
GeneisisII [21]	±20°	±20°	–

-: No Varus/valgus constraint,^a^:Not reported

### Surgical procedure

The FNK PS-TKA is used for patients with anterior cruciate ligament (ACL) and posterior cruciate ligament (PCL) deficiencies, a fixed flexion contracture > 15°, inadequate flexion gap, and moderately deficient MCL. All TKA procedures were performed by trained surgeons who specialized in joint replacement surgery. The femoral component was placed either parallel to the transepicondylar axis of external rotation or perpendicular to the Whiteside line. The ligaments were then balanced in both flexion and extension, and the implants were fixed with cement.

### In-vivo kinematic analysis

Two conditions including WB and NWB in deep knee bending were adopted in this study because previous studies suggest that these conditions affect the kinematics of the post-cam contact and posterior femoral translation. In WB deep knee flexion assessment, the patients performed sequential deep knee bends (i.e., squats), from 0° to maximum flexion under fluoroscopic monitoring in the sagittal plane. Conversely, in the NWB knee flexion assessment, the patient sat on a chair and was asked to perform active assisted knee flexion. We assisted the patient in knee bending to perform measurements on the flat panel with the heel supported.

This flexion motion was recorded as sequential digital radiographic images (2048 × 2048 bits/pixels, 7.5-Hz serial images registered in the DICOM format) using a 14-in. flat panel fluoroscopy-based detection system (Ultimax 80, Toshiba, Japan). The spatial position and orientation of the TKA components were registered using a previously described technique [12, 13, 22] Knee motion was quantified to an accuracy of 0.5° or less for rotation, and 0.4 mm or less for translation [12]. For analysis, we quantified the range of motion, axial rotation of the femoral component relative to the tibial component, anteroposterior translation of the nearest point between the medial and lateral femoral components and the tibial polyethylene insert, and the angle of post-cam engagement. The center of gravity of the femoral implant defined the origin of its coordinate system, while the center of the tibial tray defined the origin of the tibial component. Axial femoral rotation was positive for external rotation and negative for internal rotation. The center of quasi-contact at the nearest point of contact between the medial and lateral sides of the femoral component and the tibial insert was identified by calculating the shortest distance between the surfaces of the CAD models. An anterior position of the femoral component to the tibia was indicated as positive, while a posterior position was indicated as negative. The angle of post-cam engagement was identified by measuring the distance between the femoral cam and the tibial post on sequential 3-D fluoroscopic images of knee motion, with a distance < 0.5 mm defining the point of engagement, and the corresponding knee angle was registered.

### Statistical analysis

The difference in the ranges of motion under WB and NWB conditions was evaluated by paired *t*-tests. The relationship between the angles of the initial post-cam engagement and maximum knee flexion was evaluated using Pearson’s correlation. A *p* < 0.05 was considered statistically significant for all tests. All statistical analyses were performed using SPSS for Windows, Version 21 (SPSS, Chicago, USA).

## Results

### Radiographic component position

The Knee Society roentgenographic evaluation [23] is shown in Table 2.

**Table 2:** The knee Society roentgenographic evaluation

Mean ± SD (degrees)				
	α angle	β angle	γ angle	σ angle
Component alignment	95.6 ± 2.3	89.5 ± 2.6	1.8 ± 2.1	84.6 ± 3.6

### Range of motion

The relative angle between the femoral and tibial components is shown in Table 3. There were no significant differences in the angle of extension between WB and NWB, while the maximum-flexion was significantly greater under NWB than WB (*P* = 0.04).

**Table 3:** Average ranged of motion under WB and NWB conditions

	Flexion angle (degree)	Mean ± SD (range)
	Full Extension	Max Flexion
WB	−8.1 ± 8.8(−23.1–7.5°)	101.9 ± 11.6(78.1–120.2)
NWB	−7.5 ± 5.5(−17.5–2.1)	111.8 ± 6.2(101.8–125.6)

### Femoral axial rotation

**Fig. 2:**
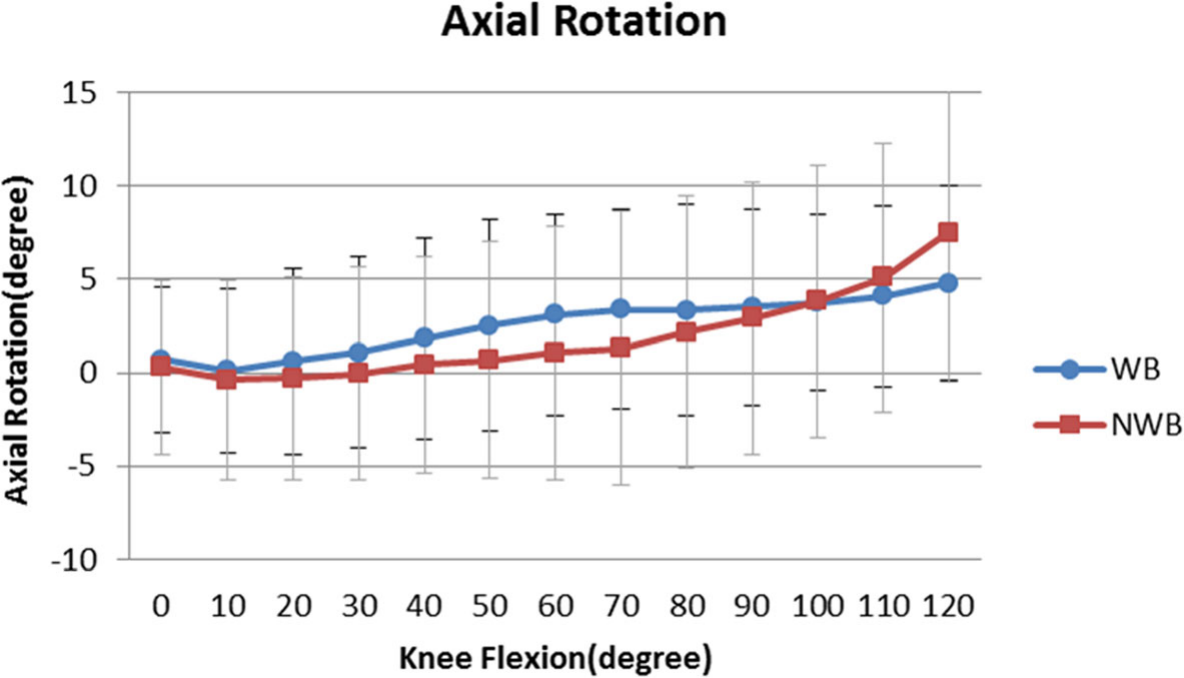
Mean femoral axial rotation relative to the tibia under WB and NWB conditions. There were no significant differences between the two conditions

The femoral component exhibited a gradual external rotation during maximum knee flexion under both WB and NWB conditions (Fig. 2.). During knee flexion from 0° to 120°, the angle of external rotation increased from 0.7 ± 3.9° to 4.8 ± 5.2° under WB and from 0.3 ± 4.7° to 6.2 ± 5.9° under NWB. There were no significant differences in the angle of external rotation between WB and NWB conditions.

### Anteroposterior translation

**Fig. 3:**
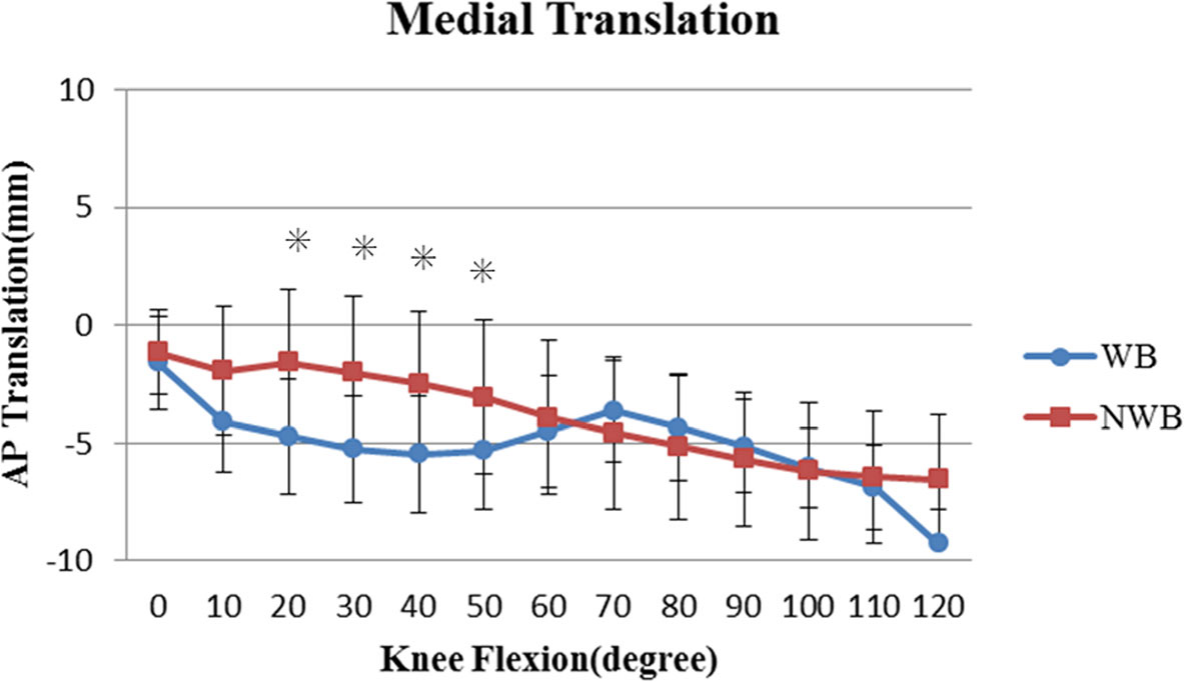
Mean anteroposterior translation of the medial femorotibial contact point under WB and NWB conditions. Asterisk indicates *P* < 0.05

**Fig. 4:**
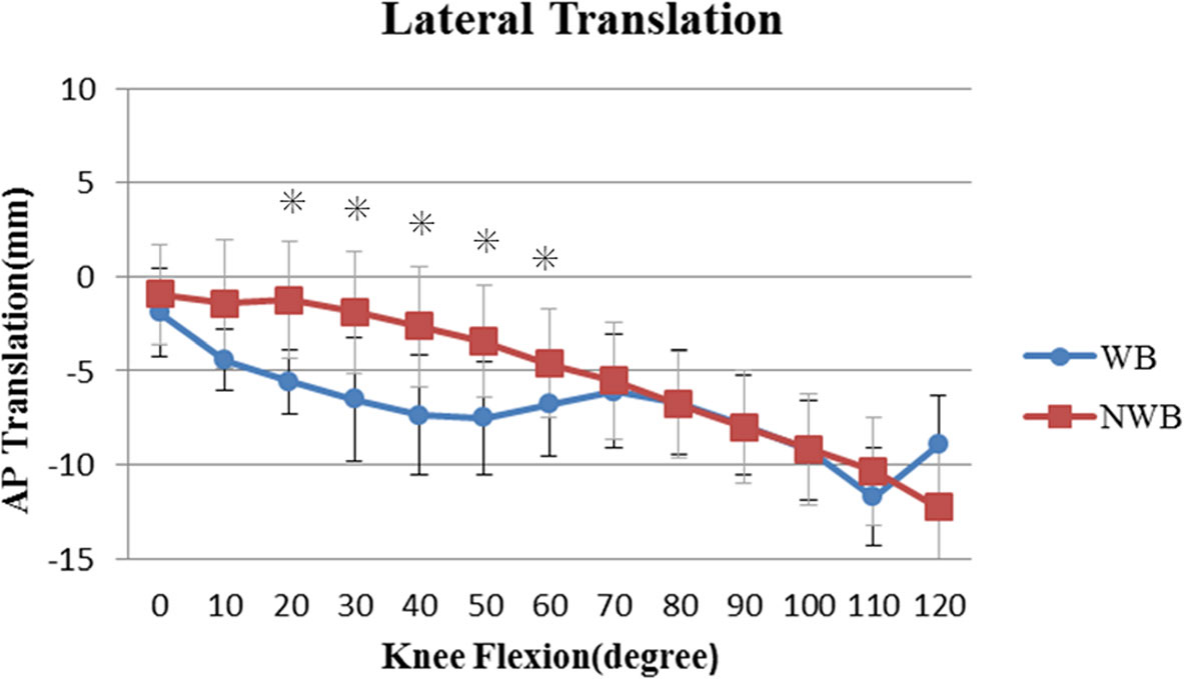
Mean anteroposterior translation of the lateral femorotibial contact point under WB and NWB conditions. Asterisk indicates *P* < 0.05

The anteroposterior translation of the femoral component relative to the tibial component in WB and NWB is shown in Figs. 3 and 4, respectively. In both, the contact point translated posteriorly from an initial position, with increasing flexion.

During the mid-flexion range, the contact point was significantly more anterior under NWB than under WB condition at both, the medial and lateral sides (*P* < 0.05). Thereafter, for deep knee flexion, there was no significant difference in contact points between the WB and NWB conditions.

### Post-cam engagement

**Fig. 5:**
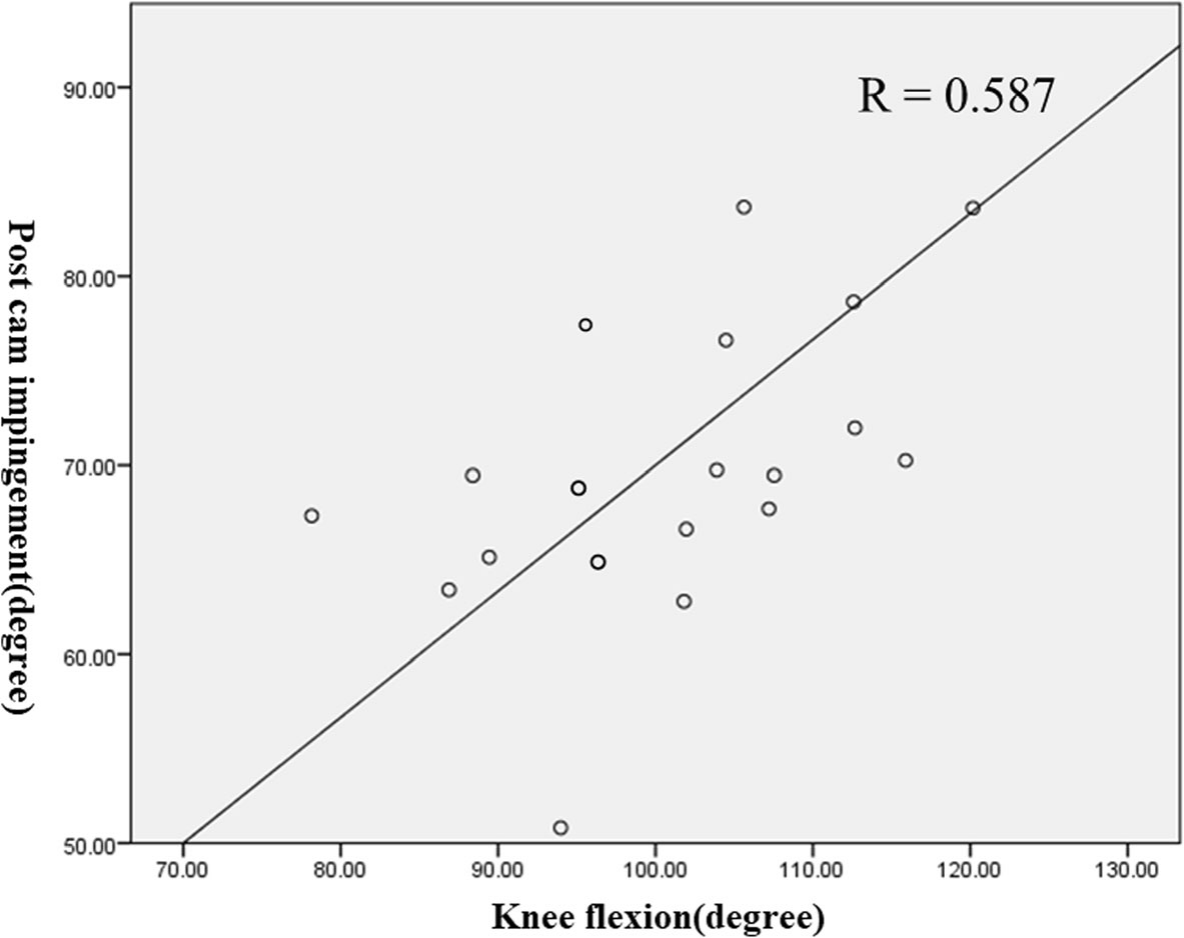
Correlation between the cam-post engagement angle and maximum flexion of the knee under WB condition (Pearson, R = 0.587, *P*<0.05)

Post-cam engagement was observed in all patients. Flexion angle of the initial post-cam engagement is shown in Table 4. Therefore, the initial post-cam engagement occurred significantly earlier in NWB than in WB (*P* = 0.04). A significant correlation between the angles of initial post-cam contact and the maximum knee flexion available was identified in WB (Fig. 5; R = 0.587, *P* = 0.02), while no observable correlation was noted in NWB (R = 0.196).

**Table 4:** Initial post cam engagement

Mean ± SD (range)	
	Flexion angle (range)
WB	61.9 ± 15.9°(50.8–83.7°)*
NWB	57.5 ± 16.0°(39.8–84.6°)*

*WB* Weight Bearing, *NWB* Non weight bearing

Asterisk indicates *P* < 0.05

## Discussion

This semi-constrained PS system reproducibly exhibited a mild external rotation with smooth posterior rolling back of the femoral condyles during deep knee bending in both WB and NWB conditions. These kinematics were similar to that of the standard PS TKAs [9–11, 24–29]. Furthermore, post-cam engagement occurred in a relatively early phase of flexion, which contributed to the reproducible femoral roll-back. To our knowledge, none of the previous studies have analyzed the effects of a large tibial post and high femoral cam on the kinematics of the TKA mechanism. Semi-constrained FNK PS-TKA demonstrated a natural knee bending in 3-D kinematics.

Regarding the maximum flexion angle, a significant difference was found between the WB and NWB conditions. A previous study [28, 29] reported that the maximum flexion angle for PS TKA was reduced under WB compared with that under NWB, which is consistent with the finding in the present study. We suggest that complex interactions in dynamic muscle forces, soft tissue constraints, and articular congruity are involved in the reduction of maximum flexion under the WB condition.

There are several studies on deep knee bending using PS-TKA under WB conditions for determining the femoral axial rotation [9–11, 22, 28, 29]. Here, a mean femoral axial rotation of 4.8° and 6.2° under WB and NWB conditions, respectively, was noted at maximum flexion. No significant difference in flexion was observed between the two conditions. Shimizu et al. studied femoral axial rotation under WB and NWB conditions with the Nexgen LPS implant; no significant difference in flexion was noted between the two conditions [10]. They suggested that the flat in the flat post-cam design might prevent a greater rotation under WB conditions and that the smaller post-cam contact force contributed to greater rotation under NWB conditions. While this FNK-PS design also featured a flat-on-flat post, it exhibited a moderate axial rotation during deep knee bending with no significant difference of the angle, similar to the previous study for Nexgen LPS [10]. The semi-constrained large post-cam mechanism did not interfere with the axial femoral rotation during the deep knee flexion.

During mid-flexion, the medial and lateral contact points were located significantly more anteriorly under NWB conditions, than under WB conditions, from 20° to 80° flexion (medial contact point: 20° to 50°, lateral contact point: 20° to 60°, [Figs. 3 and 4, respectively]; *P* < 0.05). The tibiofemoral contact point at mid-flexion was more anterior under NWB conditions, and this could be attributed to the patellar ligament force arising from the ACL and PCL deficiencies [26, 30, 31].

Femoral posterior translation occurred only after post-cam engagement at approximately 60° under the NWB conditions. However, In one study, post-cam engagement occurred significantly earlier under NWB conditions [10]. It was suggested that both condyles were located about 5 mm more anteriorly under NWB condition at the initial post-cam engagement. Our data showed a similar trend. Dennis et al. suggested that the significantly earlier post-cam engagement may be attributable to muscle force. Under WB, during early flexion, the patellar ligament pulls the tibia anteriorly due to the absence of the ACL. However, this process is reversed after 45° to 60° of flexion; the patellar ligament tends to push the tibia posteriorly due to the absence of the PCL [30]. In the current study, post-cam engagement was observed at a mean flexion angle of 61.9 ± 15.9° under WB and 57.5 ± 16.0° under NWB conditions; this engagement occurred earlier than that observed for Nexgen LPS [10, 24] . The force of quadriceps on the femur could cause the initial posterior translation of the femur before the post-cam engagement especially in WB condition. Then, the post-cam engagement increases the posterior femoral translation and enhances the knee flexion [10, 24, 27, 29]. These data also suggest a correlation between the initial post-cam engagement angle and the maximum flexion angle under WB conditions (Fig. 5).

This study has some limitations. Firstly, the number of cases was limited; only 15 cases of 19 knees were included. Secondly, a single type of semi-constrained PS prosthesis was evaluated. Thirdly, we focused only on deep knee bending. Fourth, the FNK PS system has a relatively high varus-valgus stability; however, this study did not evaluate the varus-valgus angle in each flexion angle. Finally, the contact area and stress force of the post-cam was not directly evaluated in our study. Nevertheless, the current study theoretically supports the previously reported clinical outcomes of the FNK PS-TKA, with a good range of motion and recovery of the postoperative quadriceps and hamstring power [5, 6].

## Conclusions

In conclusion, our findings explain the in vivo deep knee bending kinematics and cam-post engagement of the semi-constrained PS prostheses. The large tibial post and high femoral cam were engaged in the early phase of flexion, assisting consistent femoral roll-back with moderate axial rotation. These kinematics were in line with the development concept of the prosthesis, which provides natural kinematics compatible with a stable knee for cases of severe deformities, inadequate flexion gaps, and unbalanced knees.

## Data Availability

The datasets used and/or analysed during the current study available from the corresponding author on reasonable request.

## Abbreviations

ACL: Anterior cruciate ligament
AP: Anteroposterior
CAD: Computer-aided design
D: Dimensional
FNK: Flexible Nichidai Knee system
FTA: Femorotibial angle
MCL: Medial collateral ligament
PCL: Posterior cruciate ligament
PS: Posterior stabilized total knee arthroplasty
TKA: Total knee arthroplasty
WB: Weight-bearing
